# Stupidities

**Published:** 1873-03

**Authors:** 


### STUPIDITIES.

It is really a great wonder that everybody in the world is not dead and buried, and the world itself used up entirely, if the thousandth part of what is told us is true about microscopical and other "discoveries" so called. One man will have it that the glorious union over which the stripes and stars float so proudly will soon become depopulated, because respectable people don't have more children; another has discovered myriads of bugs in the chatelaines and waterfalls of the ladies, boring into their skulls and sucking out all the remaining brains of the dear delightfuls.

A German *savan* now tells us that every sip of tea we take is full of oily globules which get into the lungs direct, weakens them, sets up a cough, and the person dies of consumption. Another man has found that the purest spring water, clear as crystal to all appearance, if let alone will deposit a sediment which generates typhoid fever, hence he proposes that everybody shall quit drinking water.

Another one says that bread has so much lime in it that it is turning us all to bone, and makes us stiff in the joints, that being the reason we have no lithe, sprightly old men now-a-days; hence we are full of limps and rheumatics long before our time, therefore we had better quit eating bread altogether, and live on rice and sago and tapioca.

The water-cure folk assure us that pork and beans and ham and eggs are full of abominable trichina, and that if one is swallowed and gets fairly nestled into the system that he, she or it will breed a million more in a short time; and that roast beef has juvenile tape worms in it.

And here come Tom, Dick, and Harry, all in a row, loaded down with microscopes and spy-glasses which show as plain as day that the air is swarming with living monsters and putrid poisons, which fly into the mouth and crawl up the nose and creep into the ear, hence it is death to breathe such pestilential air, and that the best way is to keep the mouth shut, plug up the nose, and ram cotton into the ears.

Ever so many learned professional gentlemen have been torturing poor figures for years to make them tell the stupendous fib that everybody is either crazy or soon will be; that the annual increase is ten per cent., consequently in eleven years everybody will be crazy, and more too.

The fact is, the people who spend their time in hatching out these tom-fooleries, ought to be put to work and be made to earn an honest living. One fellow, down east, is all the time complaining and worrying over the fact that the Irish have too many babies, and respectable people won't have enough. He does not tell us whether he has done his full share in populating, but the presumption is, he isn't able to do a single solitary thing in that line, and he got mad about it. This whole thing can be remedied in a generation by just making

### A SWAP

with the Paddies. Give them all the property, and let "respectable people" and the buggy philosophers go to work and dig all the canals, and lay all the railroads, and do all the cooking and washing and other hard work about our houses; the certain result will be that these last will soon begin to have more children than they will know what to do with, while the jolly sons of "St. Patrick's day in the morning" will instinctively return to their native and dearly loved occupation of breaking each others skulls, until not a solitary Kilkenny cat of them is left to keep his own wake.

This world has been pretty well taken care of for some thousands of years, increasing in comfort and wealth and life, the average length of which last has doubled within two centuries, and the population increased perhaps three fold, and the presumption is, that the Great Maker of all will so arrange all the antagonistic forces of life for the future, as eventually to make "the wilderness and solitary place to be glad, and the desert shall rejoice and blossom as the rose," and the race be happy still.

